# Association between Ebola prevention training and prevention-related cognitive and behavioral factors among Chinese expatriates in the Democratic Republic of the Congo: a cross-sectional study informed by the RANAS framework

**DOI:** 10.3389/fpubh.2026.1979792

**Published:** 2026-10-07

**Authors:** Hao Xiao, Guangyuan Liu, Hengbo Gao, Xiaoling Zhang

**Affiliations:** 1 Department of Emergency, The Second Hospital of Hebei Medical University, Shijiazhuang, China; 2 Department of Orthopedics-II, The Third Hospital of Shijiazhuang, Shijiazhuang, China; 3 Department of Gynecology, The Second Hospital of Hebei Medical University, Shijiazhuang, China

**Keywords:** Chinese expatriates, Ebola prevention training, Ebola virus disease, RANAS, risk perception, self-regulation

## Abstract

**Objective:**

This study aimed to explore the associations between Ebola prevention training experience and multiple prevention-related outcomes (infectious disease knowledge, risk perception, protective attitudes, and psychological self-regulation) among Chinese expatriate workers in the Democratic Republic of the Congo (DRC), and to further assess potential ceiling effects and whether educational attainment modified these associations.

**Methods:**

Based on the Risk-Attitude-Norms-Ability-Self-regulation (RANAS) framework, a cross-sectional questionnaire survey was conducted among 951 Chinese expatriates in the DRC. Mann–Whitney U test and χ^2^ test were applied for univariate comparisons. General linear models (GLMs) adjusted for age, educational attainment, and residence duration in the DRC were constructed to evaluate independent associations between training experience and each outcome. Ceiling effects were quantitatively described, and sensitivity analyses excluding maximum-score participants were performed to assess potential analytical bias. Two-way interaction terms were further incorporated to test the effect modification of educational attainment.

**Results:**

Adjusted GLM results showed that participants with training experience exhibited significantly higher adjusted marginal means in basic knowledge, prevention-and-response knowledge, and psychological self-regulation (*p* < 0.05). No significant between-group differences were observed in risk perception and protective attitudes. Obvious ceiling effects existed in perceptual and attitudinal indicators, with 66.67% of participants achieving full scores in risk perception and 62.99% in protective attitudes. Sensitivity analyses indicated that ceiling effects might mask potential associations for psychological and behavioral outcomes and cause instability in the association of prevention-and-response knowledge. Significant educational modification effects were detected for the associations of training with basic knowledge and psychological self-regulation (*p* < 0.05). Stratified analyses further showed that positive associations were observed among participants with high-school education or below and those with junior-college/bachelor-level education, but not among those with postgraduate education or above.

**Conclusion:**

Ebola prevention training experience was independently associated with higher epidemic-prevention knowledge scores and better psychological self-regulation, while associations with risk perception and protective attitudes were substantially constrained by ceiling effects. The associations between training and health outcomes are heterogeneous across educational strata. Targeted and stratified health education strategies, rather than uniform training schemes, should be adopted for overseas populations to better tailor infectious-disease prevention training programs to heterogeneous overseas populations.

## 1 Introduction

Ebola virus disease (EVD) is a severe acute infectious disease caused by viruses of the genus Orthoebolavirus. It is characterized by rapid disease progression, high case-fatality rates, and substantial public health consequences, and remains one of the most concerning emerging infectious diseases worldwide (1). Since its first identification in 1976, recurrent EVD outbreaks have occurred primarily in Central and West Africa. Among affected countries, the Democratic Republic of the Congo (DRC) has experienced multiple outbreaks caused by different Ebola virus species and continues to face a persistent risk of recurrent transmission (2). In May 2026, an outbreak of Bundibugyo ebolavirus disease occurred in the DRC and was declared a Public Health Emergency of International Concern (PHEIC) by the World Health Organization (WHO) (3–5), highlighting the continued threat posed by EVD to both local communities and overseas populations (4, 6). Against this backdrop, the Chinese Medical Team to the DRC has delivered standardized Ebola prevention training for Chinese expatriates stationed in the DRC, alongside a questionnaire survey. This work aims to assess the current level of Ebola prevention-related literacy among overseas staff and examine its association with previous training experience, to provide evidence for improving public health protection among Chinese expatriates overseas.

Previous studies have demonstrated that Ebola-related protective behaviors are related not only to individuals’ knowledge of disease transmission routes and protective measures, but may also be influenced by risk perception, protective attitudes, self-regulation and other psychobehavioral factors (7). Standardized Ebola prevention training has therefore become an essential strategy for improving infection prevention and control capacity among populations living in endemic regions. Existing studies have primarily examined Ebola prevention training among healthcare workers, emergency response personnel, and local communities affected by Ebola outbreaks (8). However, evidence regarding Chinese expatriates residing in endemic countries remains scarce. In addition, populations with different educational backgrounds may differ in knowledge base, access to health-related information and health-behavior-related competencies. Evidence regarding whether the association between training experience and prevention-related indicators varies across educational attainment remains scarce.

The Risk–Attitude–Norms–Ability–Self-regulation (RANAS) is a theoretical framework centered on health-behavior change, which explains the formation and change of health behaviors from the perspectives of risk, attitude, social norms, ability and self-regulation (9). This framework has been applied to multiple research fields of health-behavior studies, including drinking-water hygiene, hand hygiene, vaccination, and infectious disease prevention and control (10). Compared with simple assessment of disease-related knowledge, constructing multi-dimensional measurements based on RANAS-related theoretical factors helps to understand health-behavior-related characteristics from different dimensions including knowledge, risk perception, attitude and self-regulation (11). However, there is still a lack of studies on Ebola prevention and control training targeting Chinese expatriates in the DRC, as well as evidence regarding whether educational attainment modifies the association between training experience and prevention-related indicators.

Therefore, this study enrolled Chinese expatriates in the Democratic Republic of the Congo as research subjects. A multi-dimensional evaluation system was constructed based on the RANAS theoretical framework to compare differences in basic knowledge, prevention-and-response knowledge, risk perception, psychological self-regulation and protective attitudes between participants who received standardized Ebola prevention and control training and those who did not. After adjusting for age, educational attainment and duration of stay in the DRC, we analyzed the association between training experience and the above-mentioned indicators. Meanwhile, by testing the interaction between training experience and educational attainment, we further explored whether educational attainment modifies the association between training experience and relevant outcomes, so as to provide references for optimizing infectious disease health education for Chinese expatriates overseas.

## 2 Methods

### 2.1 Study design and participants

This cross-sectional observational study was conducted against the background of the Ebola outbreak reported in the DRC in May 2026. In response to the outbreak, the 24th Chinese Medical Team to the DRC organized standardized Ebola prevention training for Chinese expatriates and simultaneously conducted an anonymous online questionnaire survey to examine the association between training experience and prevention-related outcomes. Figure 1 illustrates the location of the 2026 Bundibugyo ebolavirus outbreak and Chinese medical team sites in the DRC.

**Figure 1:**
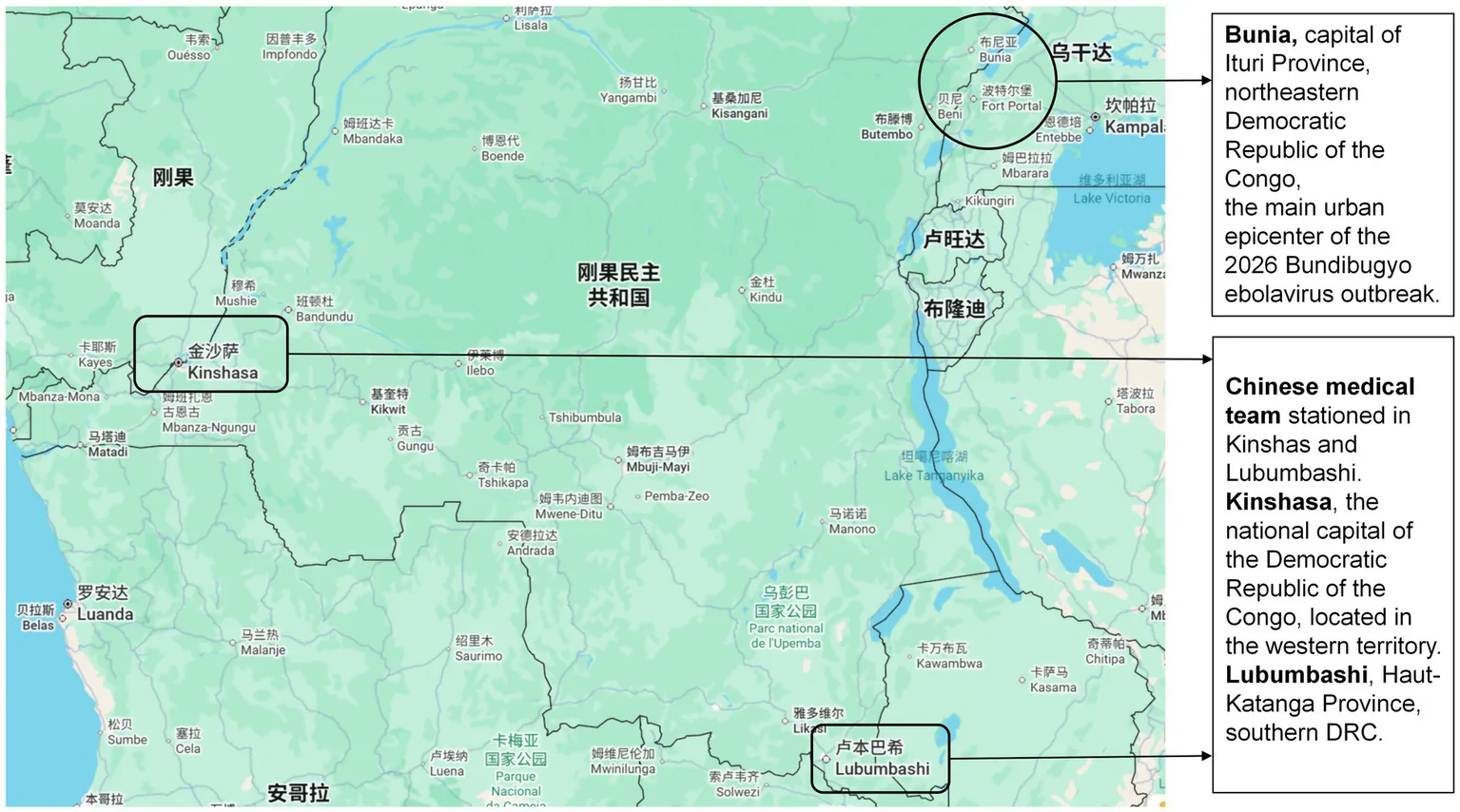
Map of the DRC, showing Bunia (epicenter of the Ebola outbreak), Kinshasa (national capital), and Lubumbashi in southern DRC, where Chinese expatriate workers and Chinese-medical-team members were stationed.

Participant recruitment and questionnaire survey were carried out from 2 June 2026 to 7 June 2026 using convenience sampling targeting Chinese-funded enterprises in the DRC. Eligible participants were Chinese expatriates working or living in the DRC. Inclusion criteria: ① aged ≥ 18 years; ② continuous residence in the DRC for ≥ 1 month; ③ capable of independently reading and completing the electronic questionnaire; ④ reading informed-consent information and voluntarily participating in the study. Exclusion criteria: ① extensive missing data in demographic information or core assessment dimensions; ② obvious logical contradictions in responses; ③ questionnaire completion time < 60 s with obvious low-quality-response features; ④ repeatedly-submitted questionnaires. A total of 966 questionnaires were collected. Fifteen unqualified questionnaires were excluded according to pre-specified data quality-control criteria, and 951 valid questionnaires were finally included, with an effective response rate of 98.4%. Among them, 190 participants who reported having received the standardized training organized by the 24th Chinese Medical Team to the DRC were assigned to the training group; the remaining 761 participants without such standardized training experience were assigned to the non-training group. Figure 2 displays the questionnaire-recruitment workflow and on-site-training photographs of this cross-sectional survey among local Chinese expatriates.

**Figure 2:**
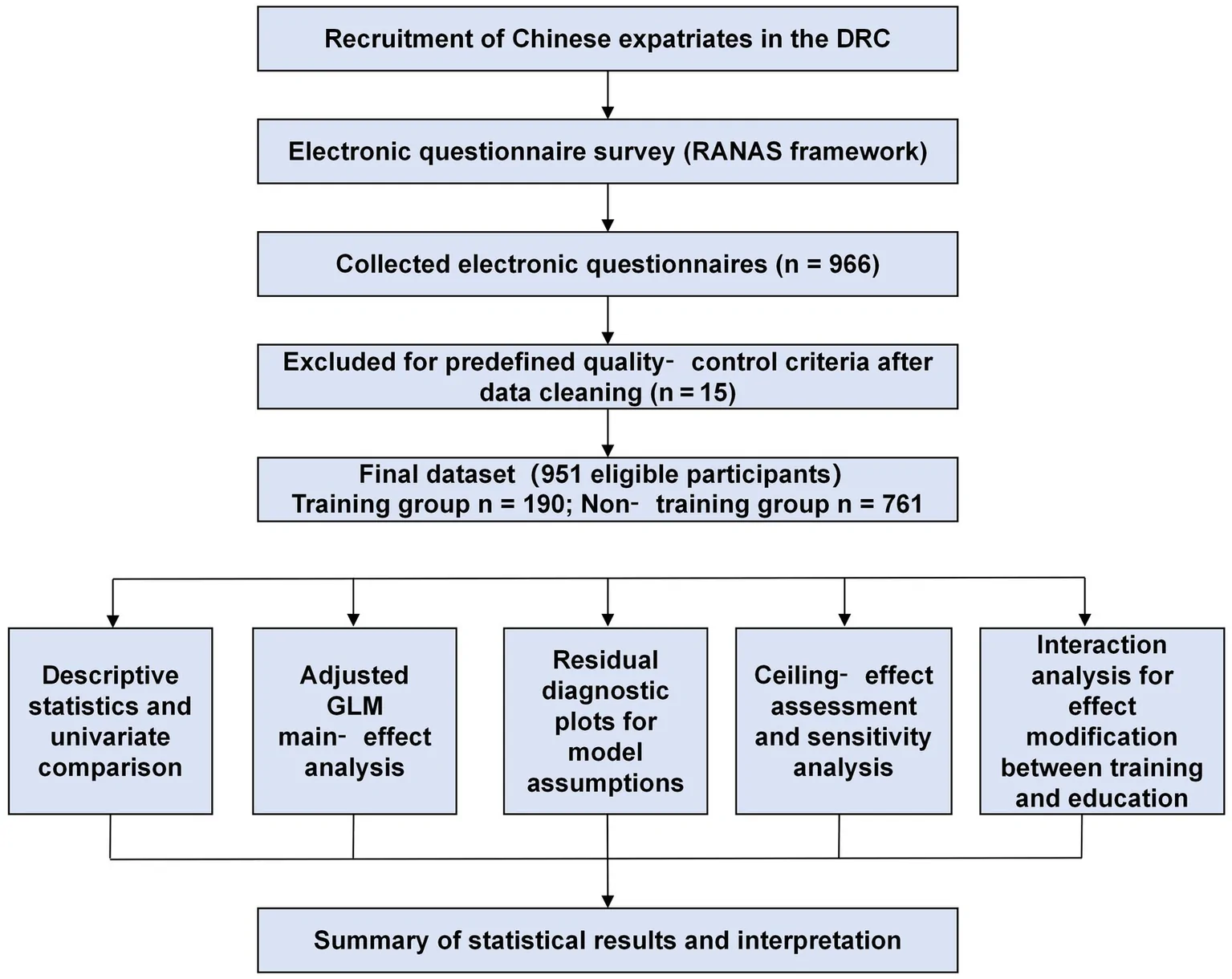
Work-flow diagram of participant recruitment and statistical analysis for this cross-sectional study among Chinese expatriates in the DRC. In total, 966 questionnaires were collected; 15 were excluded after data cleaning and quality control, leaving 951 eligible participants for final analysis. Subgroup sample sizes for training and non-training groups are reported in the main text and Supplementary Table S-1. The training group and non-training group were not formed by random allocation but divided according to self-reported actual training experience. Therefore, this study mainly evaluated statistical associations between previous training experience and assessment indicators, and inter-group differences should not be directly interpreted as causal effects attributable to training.

### 2.2 Questionnaire development and data collection

The questionnaire was independently developed by the 24th Chinese Medical Team to the DRC adapting to local conditions. Items were designed referring to the Ebola Virus Disease Prevention and Control Protocol (2026 Edition), domestic and international studies on infectious-disease-related health behaviors among overseas-labor populations, and the RANAS framework. Before formal survey implementation, 30 local Chinese residents were enrolled for a pilot survey via convenience sampling. Item wording, professional terminology, and comprehensibility of scenario-based questions were revised according to participant feedback. Three infectious-disease specialists and two epidemiologists then performed content-validity review; items were further adjusted based on expert comments to generate the formal questionnaire. The questionnaire contained two parts: general demographic information and Ebola-prevention-related assessments. Demographic variables included sex, age, educational attainment, occupation, duration of residence in the DRC, and body mass index (BMI). Five assessment domains were constructed with reference to the RANAS framework: basic knowledge, prevention-and-response knowledge, risk perception, psychological self-regulation, and protective attitudes.

Data were collected via the anonymous online questionnaire platform (Wenjuanxing). No personally identifiable information was collected or retained. Before filling in the questionnaire, all participants were informed of study objectives, voluntary-participation principles, data-confidentiality rules, and the right to withdraw without adverse consequences. Electronic informed consent was obtained prior to questionnaire completion. Each IP address was limited to one submission. Two investigators independently reviewed and verified raw data before statistical analysis. The standardized offline training was delivered face-to-face by professionals from the 24th Chinese Medical Team to the DRC, each session lasting approximately 3 h.

#### 2.2.1 Content validity and internal-consistency evaluation

The draft questionnaire underwent content-validity review by three infectious-disease specialists and two epidemiologists. Internal-consistency analysis was conducted for the three Likert-scale-based domains using formal-survey data. Cronbach’s *α* coefficients for risk perception, psychological self-regulation, and protective attitudes were 0.732, 0.788, and 0.910 respectively, indicating acceptable internal consistency.

### 2.3 Study variables

Table 1 summarizes item counts, response formats, scoring rules, and score ranges for each RANAS-based domain.

**Table 1:** Basic information of each dimension of the RANAS-based Ebola-prevention questionnaire.

Domain	No. of items	Response format	Scoring method	Score range
Basic knowledge	10	Correct/Incorrect	Correct = 1; Incorrect/Unknown = 0	0–10
Prevention-and-response knowledge	10	Correct/Incorrect	Correct = 1; Incorrect/Unknown = 0	0–10
Risk perception	6	5-point Likert scale	Sum of item scores	6–30
Psychological self-regulation	10	5-point Likert scale	Sum of item scores	10–50
Protective attitudes	8	5-point Likert scale	Sum of item scores	8–40

Basic knowledge: 10 objective single-choice items (correct = 1 point; incorrect = 0 point; total 0–10).

Prevention-and-response knowledge: 10 scenario-based objective multiple-choice items (correct = 1 point; incorrect = 0 point; total 0–10).

Risk perception: six 5-point Likert-scale items (total 6–30).

Psychological self-regulation: ten 5-point Likert-scale items (total 10–50).

Protective attitudes: eight 5-point Likert-scale items (total 8–40).

Total scores of each domain were calculated and served as outcome indicators for subsequent analyses.

### 2.4 Statistical analysis

All statistical analyses were performed using IBM SPSS Statistics version 26.0 (IBM Corp., Armonk, NY, USA). Continuous variables were reported as mean ± standard deviation or median (P_25_, P_75_) depending on distribution; categorical variables were described as counts and percentages. Baseline characteristics between training and non-training groups were compared using *χ^2^* tests or corresponding non-parametric tests. Two-tailed *P*<0.05 was defined as the threshold of statistical significance. Univariate inter-group comparisons for five outcome-scale scores were performed with the Mann–Whitney U test; χ^2^ tests were used to compare maximum-score proportions between groups. These univariate analyses were not adjusted for confounders; primary study conclusions were derived from multivariate generalized-linear-model results.

Covariates with imbalanced inter-group distributions, including age (≤ 30, 31–40, 41–50, > 50 years), educational attainment (high-school or below, junior-college/bachelor’s degree, postgraduate or above), and duration of residence in the DRC (≤ 6, 7–36, > 36 months), were adjusted in the models. Multivariable GLMs with an identity link function were fitted, taking domain total scores as outcomes and training-participation status as the main explanatory variable. Adjusted estimated marginal means (EMMs) with 95% confidence intervals (95% CI), F-statistics and *p*-values were reported, and results were interpreted as statistical associations rather than causal effects.

Potential ceiling effects were assessed by calculating the proportion of participants achieving theoretical maximum scores and inspecting residual P–P plots. Sensitivity analyses were performed by excluding participants with maximum scores for each outcome and refitting GLMs, whose results served only as auxiliary evidence. The interaction term “training experience × educational attainment” was further included to test effect modification. Estimated marginal means for each education-training combination were presented in Supplementary Table S-1 for outcomes with significant interaction (*p* < 0.05).

### 2.5 Ethics approval

The study protocol was approved by the Research Ethics Committee of the Second Hospital of Hebei Medical University (Approval No. 2026-R879). All procedures involving human participants were conducted in accordance with the ethical principles of the Declaration of Helsinki. Electronic informed consent was obtained from all participants before participation in the survey.

## 3 Results

### 3.1 Participant characteristics

A total of 951 Chinese expatriates residing in the DRC were included in this study, of whom 190 (19.98%) had previously received Ebola prevention training organized by the 24th Chinese Medical Team and 761 (80.02%) had not. The baseline characteristics of the participants are presented in Table 2. Significant differences were observed between the trained and non-training groups with respect to age, educational attainment, and duration of residence in the DRC (all *p* < 0.05), whereas no significant differences were found in sex, BMI, or occupation (all *p* > 0.05). Therefore, these variables were included as covariates in the subsequent multivariable generalized linear models.

**Table 2:** Baseline characteristics of participants according to Ebola prevention training status.

Variables	Category	Non-training group (*n* = 761)	Training group (*n* = 190)	*χ^2^/Z*	*p*-value
Sex	Male	734 (96.45%)	180 (94.74%)	1.196	0.274
	Female	27 (3.55%)	10 (5.26%)		
Age(years)	≤30	133 (17.48%)	64 (33.68%)	32.033	<0.001
	31–40	230 (30.22%)	64 (33.68%)		
	41–50	248 (32.59%)	39 (20.53%)		
	>50	150 (19.71%)	23 (12.11%)		
Educational attainment	High school and below	321 (42.18%)	61 (32.11%)	7.396	0.025
	Junior college/bachelor’s degree	404 (53.09%)	115 (60.53%)		
	Postgraduate and above	36 (4.73%)	14 (7.37%)		
Occupation	Self-employed and family members	79 (10.38%)	27 (14.21%)	2.251	0.134
	Employees of Chinese-funded enterprises	682 (89.62%)	163 (85.79%)		
Duration of residence (months)	≤6	281 (36.92%)	106 (55.79%)	39.564	<0.001
	7-36	252 (33.11%)	67 (35.26%)		
	>36	228 (29.96%)	17 (8.95%)		
BMI (kg/m^2^)		24.69 (22.86, 26.78)	24.99 (22.86, 26.89)	−1.075	0.282

Data are presented as n (%) or median (P_25_, P_75_). P were obtained using the χ^2^ test for categorical variables and the Mann–Whitney U test for continuous variables. Statistically significant differences (*p* < 0.05) were observed for age group, educational attainment, and duration of residence in the DRC.

### 3.2 Distribution of outcome-scale scores and ceiling phenomenon

The median and interquartile range values of five outcome scores are presented in Table 3. The Mann–Whitney U test was used for univariate between-group comparisons between the training group and non-training group. Unadjusted crude univariate analyses showed statistically significant differences in scores of basic knowledge, prevention-and-response knowledge and psychological self-regulation, whereas no significant inter-group differences were observed for risk perception and protective attitudes. According to univariate results, the median scores of risk perception and protective attitudes reached the theoretical maximum of the corresponding scales, preliminarily indicating obvious ceiling effects. We further calculated the proportion of participants achieving the theoretical maximum score for each domain, and the *χ^2^* test was applied for univariate comparison of maximum-score rates between groups (Table 4). The overall proportion of participants with maximum scores was 66.67% for risk perception and 62.99% for protective attitudes, while the proportion was relatively low for psychological self-regulation at only 3.36%. These unadjusted comparisons did not account for age, educational attainment, or duration of residence; adjusted associations are presented in Table 5.

**Table 3:** Univariate comparison of five RANAS-domain scores between the trained and untrained groups.

Outcome measures	Non-training group	Training group	*Z*	*p*-value
Basic knowledge	9.00 (8.00, 9.00)	9.00 (9.00, 10.00)	−8.452	<0.001
Prevention and response knowledge	9.00 (9.00, 10.00)	10.00 (9.00, 10.00)	−4.048	<0.001
Risk perception	30.00 (29.00, 30.00)	30.00 (28.00, 30.00)	−1.528	0.126
Psychological self-regulation	35.00 (34.00, 38.00)	39.00 (34.00, 41.00)	−5.106	<0.001
Protective attitudes	40.00 (37.00, 40.00)	40.00 (38.00, 40.00)	−0.897	0.370

Scores were presented as median (P_25_, P_75_). The Mann–Whitney U test was used for between-group comparisons. This was a univariate analysis without adjustment for confounders including age, educational attainment and residence duration in the DRC. Primary conclusions of this study were based on results from the multivariate adjusted GLM (Table 5).

**Table 4:** Univariate comparison of maximum-score proportion for each domain between the trained and non-training groups.

Outcome measures	Training group (*n* = 190) maximum-score *n* (%)	Non-training group (*n* = 761) maximum-score n (%)	Total sample (*n* = 951) maximum-score n (%)	*χ^2^*	*p*-value
Basic knowledge	80 (42.11%)	145 (19.05%)	225 (23.66%)	44.73	<0.001
Prevention and response knowledge	118 (62.11%)	355 (46.65%)	473 (49.74%)	14.53	<0.001
Risk perception	112 (58.95%)	522 (68.59%)	634 (66.67%)	6.37	0.012
Psychological self-regulation	7 (3.68%)	25 (3.29%)	32 (3.36%)	0.07	0.785
Protective attitudes	105 (55.26%)	494 (64.91%)	599 (62.99%)	6.07	0.014

Maximum-score *n* (%) refers to the number and within-group percentage of participants achieving the theoretical maximum score of each domain. Training group *n* = 190, non-training group *n* = 761, total sample *n* = 951; The χ^2^ test was applied to compare maximum-score proportions. This univariate analysis was not adjusted for confounders. Primary conclusions were derived from multivariate adjusted GLM results (Table 5).

**Table 5:** Comparison of estimated marginal means (EMM) for each outcome domain between training and non-training groups after adjustment for confounders.

Outcome domain	EMM (95% CI), training group	EMM (95% CI), non-training group	*F*(df₁,df₂)	*p*-value	Adjusted *R^2^*
Basic knowledge	9.308 (9.114–9.501)	8.642 (8.514–8.769)	*F* (1,942) = 47.068	<0.001	0.084
Prevention-and-response knowledge	9.483 (9.266–9.701)	9.191 (9.048–9.335)	*F* (1,942) = 7.156	0.008	0.029
Risk perception	28.836 (28.384–29.288)	28.725 (28.427–29.024)	*F* (1,942) = 0.235	0.628	0.008
Psychological self-regulation	38.352 (37.516–39.187)	36.445 (35.893–36.997)	*F* (1,942) = 20.643	<0.001	0.028
Protective attitudes	38.498 (37.928–39.067)	38.067 (37.690–38.443)	*F* (1,942) = 2.268	0.132	0.012

General linear model (GLM) with identity link function. Covariates adjusted included age, educational attainment, and residence duration in the DRC. Adjusted estimated marginal means, 95% CI, F-statistic, *p*-value and R^2^ were reported.

### 3.3 Adjusted associations between training experience and outcome scores

General linear models (GLM, identity link function) were constructed with adjustment for confounding factors including age, educational attainment, and duration of residence in the DRC. Adjusted estimated marginal means of each outcome stratified by training-participation status were obtained (Table 5). The results suggested positive associations of training experience with scores of basic knowledge and prevention-and-response knowledge. The adjusted estimated marginal means for these two domains were higher in the training group than in the non-training group, with statistically significant between-group differences. No statistically significant difference was observed for risk perception and protective attitudes. Participants receiving training showed significantly higher scores of psychological self-regulation compared with the non-training group. Residual P–P plots and residual-versus-fitted scatter plots (Figure 3) were generated for each main-analysis GLM. Residuals for several outcomes deviated moderately from the normality assumption. Therefore, domain-specific sensitivity analyses excluding participants with theoretical maximum scores were performed to evaluate potential bias caused by ceiling effects.

**Figure 3:**
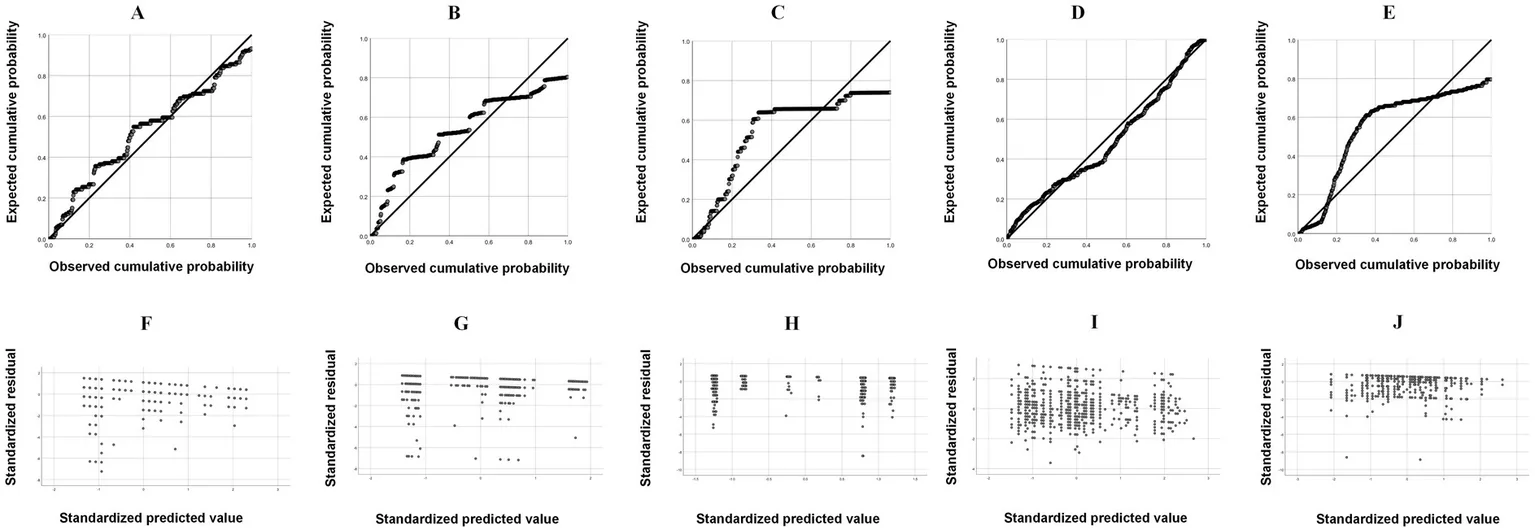
Residual diagnostic plots for five general linear regression models of Ebola-related outcome scores. **(A–E)** P–P plots of standardized residuals: **(A)** basic knowledge; **(B)** prevention-and-response knowledge; **(C)** risk perception; **(D)** psychological self-regulation; **(E)** protective attitudes. **(F–J)** Scatter plots of standardized residual versus standardized predicted value for homoscedasticity evaluation: **(F)** basic knowledge; **(G)** prevention-and-response knowledge; **(H)** risk perception; **(I)** psychological self-regulation; **(J)** protective attitudes.

### 3.4 Sensitivity analysis for ceiling-effect assessment

To evaluate the potential impact of ceiling effects on study findings, participants who obtained the theoretical maximum score for each corresponding outcome domain were excluded. General linear models (GLM) were refitted with exactly the same confounding-variable adjustment settings as adopted in the main analysis for sensitivity analysis (Table 6). After excluding maximum-score observations, statistically significant associations between training experience and higher outcome scores were detected for basic knowledge, risk perception, psychological self-regulation, and protective attitudes. The between-group association for prevention-and-response knowledge was no longer statistically significant. The primary conclusions of this study were based on the full-sample analysis. Sensitivity analysis served only as supplementary evidence to assess result robustness.

**Table 6:** Sensitivity-analysis for the association between training experience and scores after exclusion of participants with maximum scale scores.

Outcome domain	Valid sample size N after excluding maximum-score cases	EMM (95% CI), training group	EMM (95% CI), non-training group	*F*	*p*-value	Adjusted *R^2^*
Basic knowledge	726	8.726(8.493–8.958)	8.291(8.144–8.437)	13.625	<0.001	0.054
Prevention-and-response knowledge	478	8.595(8.199–8.991)	8.433(8.172–8.695)	0.65	0.42	0.009
Risk perception	317	26.721(25.737–27.706)	25.306(24.514–26.098)	9.746	0.002	0.024
Psychological self-regulation	919	37.747(36.993–38.501)	35.788(35.282–36.295)	26.787	<0.001	0.031
Protective attitudes	352	36.561(35.517–37.604)	34.218(33.471–34.966)	22.12	<0.001	0.052

For sensitivity analysis, participants achieving the maximum score for the corresponding outcome domain were excluded. General linear models (GLM) were fitted with the same set of adjusted covariates (age, educational attainment, residence duration in the DRC) as in the main analysis. The primary conclusions of this study were based on the full-sample main analysis; sensitivity analysis was applied to assess potential bias induced by ceiling effects.

Three distinct result patterns were observed across different outcomes. ① For risk perception and protective attitudes: no statistically significant association was identified in the full-sample analysis, whereas an association emerged after excluding maximum-score participants, suggesting that ceiling effects may mask the true underlying association between training experience and these outcomes. ② For basic knowledge and psychological self-regulation: statistically significant associations were observed in both the full-sample analysis and the subsample excluding maximum-score participants, indicating relatively robust associations. ③ For prevention-and-response knowledge: a statistically significant association existed in the full-sample analysis but disappeared after excluding maximum-score cases. A higher proportion of participants in the training group achieved maximum scores for this domain, and between-group score differences were concentrated at the maximum-score range of the scale. Excluding maximum-score individuals alters the composition of the analytical sample; hence, the sensitivity-analysis result for this outcome cannot override the main full-sample findings and should be interpreted with caution. In summary, sensitivity analysis served only as supplementary evidence, and the main conclusions of this study were based on the full-sample main analysis.

### 3.5 Effect-modification test of educational attainment on the association between training and outcomes

The two-way interaction term of training × educational attainment was incorporated into the GLM. Age and duration of residence in the DRC were adjusted to test whether educational attainment acted as an effect modifier (Table 7). Statistically significant interaction terms were found for basic knowledge and psychological self-regulation scores (*F* = 4.404, *p* = 0.012; *F* = 4.075, *p* = 0.017), suggesting that educational attainment modified the association between training experience and scores for these two outcomes. No statistically significant interactions were observed for risk perception, protective attitudes, and prevention-and-response knowledge. Adjusted estimated marginal means under different combinations of educational attainment and training status are shown in Supplementary Table S1. The association pattern between training experience and outcome scores varied across educational strata: higher adjusted scores in the training group were found among participants with high-school education or below, as well as those with junior-college or bachelor-level education. Such between-group difference was not observed in the postgraduate-and-above subgroup.

**Table 7:** Summary of interaction test results (training × educational attainment) for five outcome domains.

Outcome domain	*F*(df₁,df₂)	Interaction *P*-value	Adjusted *R^2^*	Effect-modification test result
Basic knowledge	*F* (1,940) = 4.404	0.012	0.091	Present
Prevention-and-response knowledge	*F* (1,940) = 1.792	0.167	0.030	Absent
Risk perception	*F* (1,940) = 0.232	0.793	0.006	Absent
Psychological self-regulation	*F* (1,940) = 4.075	0.017	0.035	Present
Protective attitudes	*F* (1,940) = 0.019	0.982	0.010	Absent

General linear models (GLM) including main effects for training, educational attainment, as well as the training × educational-level interaction term. Covariates age and residence duration in the DRC were adjusted.

## 4 Discussion

This study explored the association between previous Ebola-related health-safety training experience and multi-dimensional cognitive, psychological-behavioral indicators among Chinese expatriate workers in the DRC. After adjusting for age, educational attainment and duration of local residence, participants with training experience demonstrated higher scores for basic knowledge, prevention-and-response knowledge, and psychological self-regulation. No statistically significant between-group differences were found for risk perception and protective attitudes in the main full-sample analysis. These findings suggested that standardized training experience showed relatively clear associations with knowledge-related outcomes and psychological self-regulation, whereas no significant associations were observed for risk-perception and protective-attitude indicators in the full-sample primary analysis (12). Further analyses revealed ceiling effects for some outcome scales, and educational attainment might modify the association between training experience and scores of basic knowledge as well as psychological self-regulation (9, 13–19).

Higher scores of basic and prevention-and-response knowledge in the training group were consistent with the training content covering transmission routes of Ebola virus, personal protective measures, post-exposure management, and protective behaviors during epidemic events (20). Knowledge-related indicators had direct correspondence with training content; therefore, associations between training experience and knowledge scores were relatively easy to detect. For expatriates staying long-term in regions with ongoing Ebola epidemic threats, systematic organizational health education could help them acquire standardized information on infectious-disease prevention and reduce knowledge gaps caused by heterogeneous individual information sources (21). Nevertheless, this was a cross-sectional observational study, and training was not randomly allocated. Hence, the results could only demonstrate statistical association rather than causal effects of training (22). Baseline pre-training knowledge levels were not collected in this survey; consequently, we were unable to judge whether pre-existing between-group differences in knowledge already existed before training.

Unlike knowledge-based outcomes, among psychological-behavioral outcomes, significant association was only detected for psychological self-regulation, whereas no significant associations were observed for risk perception and protective attitudes in the primary analysis. A plausible explanation is that participants in this cohort already attained very high scores for risk perception and protective attitudes, approaching the upper limit of the measurement scale. During the Ebola epidemic outbreaks, respondents continuously obtained epidemic-related information via their employers, medical teams, mass media and other channels, which might have elevated scores for attitudinal- and risk-perception-related indicators at the time of investigation (23). Under such circumstances, measurable score differences for these two indicators between participants with and without standardized one-off training would become limited. By contrast, psychological self-regulation had a low ceiling proportion and still retained sufficient score range to detect group-level differences. The robust associations observed for knowledge outcomes and psychological self-regulation did not necessarily imply that training exerted no potential influence on risk perception and protective attitudes; instead, it indicated that different outcome domains exhibited heterogeneous sensitivity to training experience (24). Notably, psychological self-regulation exhibited a very low ceiling rate, with only 3.36% of participants achieving the maximum score. This may partly explain why a robust significant association was observed in both the main and sensitivity analyses.

Noticeable ceiling effects were identified for several outcome scales in the present investigation (25). The proportions of participants achieving maximum scores reached 66.67% for risk perception and 62.99% for protective attitudes; nearly half of participants obtained full scores for prevention-and-response knowledge, and a certain proportion of respondents also reached the maximum score for basic knowledge. Restricted score distributions might compress between-group differences and reduce the statistical power of conventional linear models to detect associations (26). We therefore performed sensitivity analyses excluding participants with maximum scale scores to evaluate the potential bias caused by ceiling effects. After exclusion of maximum-score observations, the significant association remained for basic knowledge; newly significant associations emerged for risk perception, psychological self-regulation and protective attitudes, while the association for prevention-and-response knowledge disappeared. The inconsistent changes across outcomes in sensitivity analyses suggested that ceiling effects could distort the detection of associations for certain indicators, and some findings were not fully stable. Since exclusion of maximum-score individuals altered the composition of analytical samples, sensitivity-analysis results were treated only as supplementary evidence, and the primary conclusions of this study relied on the main full-sample analysis.

Significant two-way interaction terms between training experience and educational attainment were detected for basic-knowledge and psychological-self-regulation outcomes, while no statistically significant interactions were found for the remaining three indicators. This finding indicated that educational attainment did not exert uniform effect-modification across all outcome domains (27). According to stratified adjusted estimated marginal means, within subgroups of participants with high-school-or-below education and junior-college/bachelor-level education, the training group achieved higher scores than the non-training group; such between-group difference was absent among participants at postgraduate-and-above educational stratum. Therefore, the association between training experience and relevant outcomes varied across educational strata (28). Such heterogeneity might be partly explained by disparities in prior health-related knowledge reserve and information-seeking capacity. Participants with higher educational attainment tended to have better pre-existing knowledge and multiple independent information sources, leaving limited measurable room for further improvement after training. In contrast, workers with lower educational attainment were more dependent on organizational workplace health education to acquire systematic epidemic-prevention knowledge, which made score differences more observable. It should be emphasized that we did not directly collect data on prior health-literacy status or previous relevant educational experiences; the above-mentioned interpretation remains speculative and cannot confirm the underlying mechanism of the detected interaction.

From the perspective of public-health practice, our results suggested that while maintaining standardized core knowledge for all trainees, tailored training strategies taking educational background into consideration should be considered for Chinese expatriate personnel (29). For workers with relatively weak baseline knowledge, emphasis could be placed on core content including transmission routes, personal protection and post-exposure management together with practical demonstrations. For highly educated personnel with sufficient prior knowledge, scenario-based exercises, case discussions and emergency-decision training could be added to facilitate translation of theoretical knowledge into practical emergency-response capacity. It is worth noting that the stratified training recommendation was derived from observational findings of this cross-sectional study, rather than evidence from intervention trials. This practical suggestion does not imply implementing completely separate training schemes for different educational subgroups; prospective intervention studies are still required to validate its real-world effectiveness.

These findings also have implications for occupational health education among Chinese expatriates (30). Standardized core training may provide a common foundation for infectious-disease prevention, while educationally tailored content may improve the targeting of limited health-education resources. Such approaches may be particularly relevant in overseas settings where access to timely health information and medical resources varies.

Several limitations of this study should be acknowledged. First, as a cross-sectional observational investigation without random allocation of training, causal inference cannot be established, and findings may still be subject to selection bias and residual confounding. Second, training-related information was collected via retrospective self-report; we failed to quantify training frequency, pre-training baseline cognition, content absorption and long-term knowledge retention. Third, the self-designed questionnaire was reviewed by experts for content validity and internal consistency reliability, but formal structural validity and external validity assessment were not performed. Fourth, convenience sampling was adopted, and subjects were restricted to Chinese expatriate workers in the DRC; caution should be exercised when generalizing our results to other overseas populations in different countries. Finally, prominent ceiling effects existed for several outcome scales, which constrained the detection of inter-group differences; sensitivity-analysis results also demonstrated that different indicators suffered from ceiling-related interference to varying extents.

## Data Availability

The original contributions presented in the study are included in the article/Supplementary material, further inquiries can be directed to the corresponding authors.
