# Supplementary material for: Association between Ebola prevention training and prevention-related cognitive and behavioral factors among Chinese expatriates in the Democratic Republic of the Congo: a cross-sectional study informed by the RANAS framework
Source: Front Public Health. 2026 Oct 7;14:1979792. doi: 10.3389/fpubh.2026.1979792 (PMC13683614; doi:10.3389/fpubh.2026.1979792)
Supplement: Supplementary file 1 [file Supplementary_file_1.DOCX]

Supplementary Table S‑1 Adjusted estimated marginal means (95% CI) across subgroups of educational attainment and training status

| Outcome domain | High‑school or belowTraining / Non‑training | Junior‑college / Bachelor’s degreeTraining / Non‑training | Postgraduate and aboveTraining / Non‑training |
| --- | --- | --- | --- |
| Basic knowledge | 9.174(8.879‑9.469) / 8.239(8.108‑8.371) | 9.381(9.160‑9.602) / 8.774(8.660‑8.888) | 8.903(8.290‑9.515) / 9.127(8.748‑9.505) |
| Psychological self‑regulation | 38.148(36.871‑39.424) / 35.540(34.971‑36.109) | 38.654(37.699‑39.608) / 36.683(36.190‑37.176) | 35.818(33.170‑38.467) / 38.153(36.517‑39.790) |

**Note:** GLM including main effects of training and educational level plus training × educational attainment; adjusted for age and residence duration in the DRC. Each cell shows estimated marginal mean (95% CI) for training group /non‑training group. Subgroup sample sizes: High‑school or below: training n=61, non‑training n=321; Junior‑college / Bachelor’s degree: training n=115, non‑training n=404; Postgraduate and above: training n=14, non‑training n=36.
